# MiRNA-124 is a link between measles virus persistent infection and cell division of human neuroblastoma cells

**DOI:** 10.1371/journal.pone.0187077

**Published:** 2017-10-26

**Authors:** Hila Naaman, Glenn Rall, Christine Matullo, Isana Veksler-Lublinsky, Yonat Shemer-Avni, Jacob Gopas

**Affiliations:** 1 The Shraga Segal Department of Microbiology and Immunology and Genetics, Faculty of Health Sciences Ben-Gurion University of the Negev, Beer Sheva, Israel; 2 Fox Chase Cancer Center, Blood Cell Development and Function, Philadelphia, Pennsylvania, United States of America; 3 Software and Information Systems Engineering, Ben-Gurion University of the Negev, Beer Sheva, Israel; Institut de Biologie Moleculaire et Cellulaire, FRANCE

## Abstract

Measles virus (MV) infects a variety of lymphoid and non-lymphoid peripheral organs. However, in rare cases, the virus can persistently infect cells within the central nervous system. Although some of the factors that allow MV to persist are known, the contribution of host cell-encoded microRNAs (miRNA) have not been described. MiRNAs are a class of noncoding RNAs transcribed from genomes of all multicellular organisms and some viruses, which regulate gene expression in a sequence-specific manner. We have studied the contribution of host cell-encoded miRNAs to the establishment of MV persistent infection in human neuroblastoma cells. Persistent MV infection was accompanied by differences in the expression profile and levels of several host cell-encoded microRNAs as compared to uninfected cells. MV persistence infection of a human neuroblastoma cell line (UKF-NB-MV), exhibit high miRNA-124 expression, and reduced expression of cyclin dependent kinase 6 (CDK6), a known target of miRNA-124, resulting in slower cell division but not cell death. By contrast, acute MV infection of UKF-NB cells did not result in increased miRNA-124 levels or CDK6 reduction. Ectopic overexpression of miRNA-124 affected cell viability only in UKF-NB-MV cells, causing cell death; implying that miRNA-124 over expression can sensitize cells to death only in the presence of MV persistent infection. To determine if miRNA-124 directly contributes to the establishment of MV persistence, UKF-NB cells overexpressing miRNA-124 were acutely infected, resulting in establishment of persistently infected colonies. We propose that miRNA-124 triggers a CDK6-dependent decrease in cell proliferation, which facilitates the establishment of MV persistence in neuroblastoma cells. To our knowledge, this is the first report to describe the role of a specific miRNA in MV persistence.

## Introduction

Measles virus (MV) is a member of the Paramyxoviridae family, genus Morbillivirus; it is a single-stranded, negative-sense, enveloped RNA virus. The genome is comprised of ~16,000 nucleotides, encoding eight proteins [1, 2]. Despite generally successful vaccination efforts, MV remains a major cause of preventable illness and death. It is generally presumed that immune-mediated resolution of an uncomplicated acute MV infection results in complete viral clearance. There is, however, a compelling body of evidence suggesting that MV can remain in the human population [3], and, while rare, can trigger disease long after acute infection. Most well-known of these is the ability of MV to establish long-term persistent infection in the central nervous system (CNS), leading to diseases such as subacute sclerosing panencephalitis (SSPE) and MV inclusion body encephalitis (MIBE), both of which become apparent months to years after the initial primary infection [4, 5]. Measles virus persistent infection has been studied extensively [1, 6, 7]. However, to our knowledge, there is no information available regarding the role of neuron-enriched miRNAs in facilitating MV persistence.

MicroRNAs (miRNA) are a class of ~22 nucleotide long, noncoding RNAs that are transcribed from the genomes of all multicellular organisms and some DNA viruses [8, 9]. Most RNA viruses, including MV, do not encode miRNAs [10]. Specific miRNAs have been implicated in diverse biological processes, including development, cellular differentiation, proliferation, apoptosis, and oncogenesis [11]. Individual miRNAs may regulate several hundred genes, and it is estimated that more than 30% of animal genes may be subject to miRNA control, underscoring the emerging importance of miRNAs mediated gene regulation [12]. Moreover, miRNAs are attractive therapeutic candidates due to their small size, lack of immunogenicity, and remarkable functional flexibility [13].

We explored the question of whether host-encoded miRNAs are differentially expressed in MV persistently infected cells. In particular, we investigated the role of the cellular hsa-miRNA-124, which we show to be strongly expressed in cells persistently infected with MV. MiRNA-124 is one of the best-characterized miRNAs in the CNS; it is abundantly expressed in differentiated neuronal cells and in tumors of neuronal origin [14, 15].

MV acute infection of peripheral blood lymphocytes (PBL) reduces expression of cyclin-dependent protein kinase 6 (CDK6), causing cell cycle arrest [16, 17]. CDK6 is an important regulator of cell cycle progression, regulating the G1/S transition [18–20]. The gene encoding CDK6 is also a target for miRNA-124; thus, it is well established that miRNA-124 down-regulates the expression of CDK6 [21–24]. Here, we link these findings, and show that in persistently infected UKF-NB-MV cells, compared to uninfected cells, miRNA-124 is strongly expressed, and CDK6 expression is reduced. Consequently, MV persistently infected cells exhibit slower division and sensitization to apoptosis. Similarly, miRNA-124 overexpression induces cell death in these cells, but not in uninfected UKF-NB cells. Interestingly, in cells acutely infected with the virus, we observed no increase in miRNA-124, nor reduction in CDK6, or impaired cell division. When uninfected cells were engineered to stably express miRNA-124, we attained the efficient establishment of MV persistence following acute infection.

We propose that MV persistent infection is facilitated by the down-regulation of CDK6 by miRNA-124 in neuroblastoma cells. These results might contribute to the understanding of the role of host miRNAs in inducing MV persistence in CNS derived cells, and may identify novel cellular targets to prevent or resolve persisting viral infections.

## Results

### Characterization of UKF-NB cells persistently infected with MV

**Fig 1:**
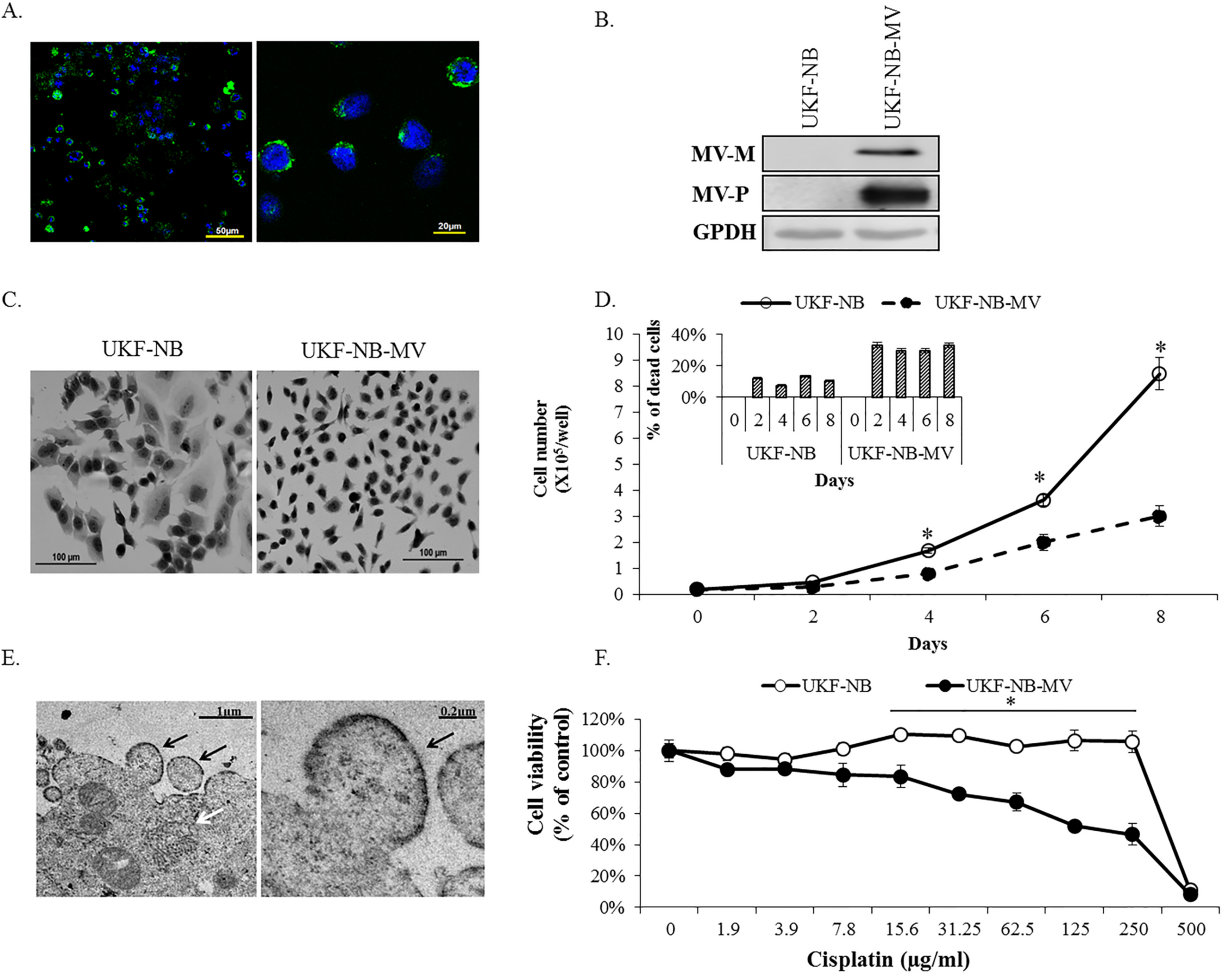
Characterization of UKF-NB-MV. (A) Confocal images of fluorescent double staining of UKF-NB-MV cells with MV-P-protein antibodies (green); nuclear staining is demonstrated with DAPI (blue). Bars indicate original image sizes. (B) MV-P and M were detected by Western blot in UKF-NB-MV cells. (C) Hematoxylin-eosin staining of UKF-NB and UKFNB-MV. Both pictures were taken at the same magnification. (D) Time curve of cell growth and death. 2x10^4^ cells/well were seeded in 6 wells plates in triplicate. The average of cell proliferation and percentage of cell death were calculated from triplicates of three independent experiments. Averages with SEs of each point are indicated for three independent experiments, each point in triplicate. T-test was performed and significance of p<0.05 is shown (*). (E) Electron Microscopy of UKF-NB-MV cells. UKF-NB-MV cells were prepared for electron microscopy by conventional techniques. MV RNP (ribonucleoprotein) assembly particles (white arrow) and a rare instance of budding virus (black arrows) are shown in two magnifications. (F) UKF-NB-MV are more sensitive to cell death as compared to uninfected UKF-NB cells. 1.5x10^5^ cells/well UKF-NB-MV or UKF-NB cells were plated. 12 hours later, cisplatin was added at different concentrations in triplicate for 48 h, and the percentage of viable cells was determined by XTT. Experiments were repeated at least three times. The percent viability of each cell type is compared to untreated cells. Averages with SEs of each point are indicated for three independent experiments, each point in triplicate. T—test was performed and significance of p<0.05 is shown (*).

The factors that allow highly cytopathic viruses to persist in some unique cell populations remain largely unknown. Thus, the study of the interplay between the MV and neurons during non-cytopathic infections is important both to refine our understanding of virus-host interactions, and to identify more targeted antiviral strategies to resolve persistent viral infections. To this end, we have studied a human neuroblastoma cell line [25], persistently infected with MV (Edmonston strain). As shown in Fig 1, the persistently infected UKF-NB-MV cells showed MV P-protein (phosphoprotein) cytoplasmic staining by immunofluorescence (Fig 1A). MV-P and MV-M (matrix) proteins were also detected by western blot (Fig 1B). UKF-NB-MV cells attached more loosely to the plastic, and differed morphologically from uninfected cells (e.g., a lower cytoplasm to nucleus ratio, denser chromatin, and more binucleated cells) (Fig 1C). UKF-NB-MV cells grew more slowly than UKF-NB cells, and had a shorter life span, as a higher percentage of dead cells were typically present in the cultures (Fig 1D). Viral ribonucleoprotein (RNP) was detected by electron microscopy in most UKF-NB-MV cells (Fig 1E), though we rarely detected budding particles from these cells, consistent with poor recovery of infectious virions from these cultures. This parallels findings from permissive primary mouse neurons, in which MV infection occurs, but does not result in release of infectious progeny, or neuronal loss [26]. Interestingly, the UKF-NB-MV line was significantly more sensitive to stress-induced cell death (e.g., cisplatin treatment) as compared to UKF-NB cells, consistent with the idea that MV may sensitize cells to death [27, 28] (Fig 1F).

### MiRNA-124 is expressed in MV persistently infected UKF-NB cells

**Fig 2:**
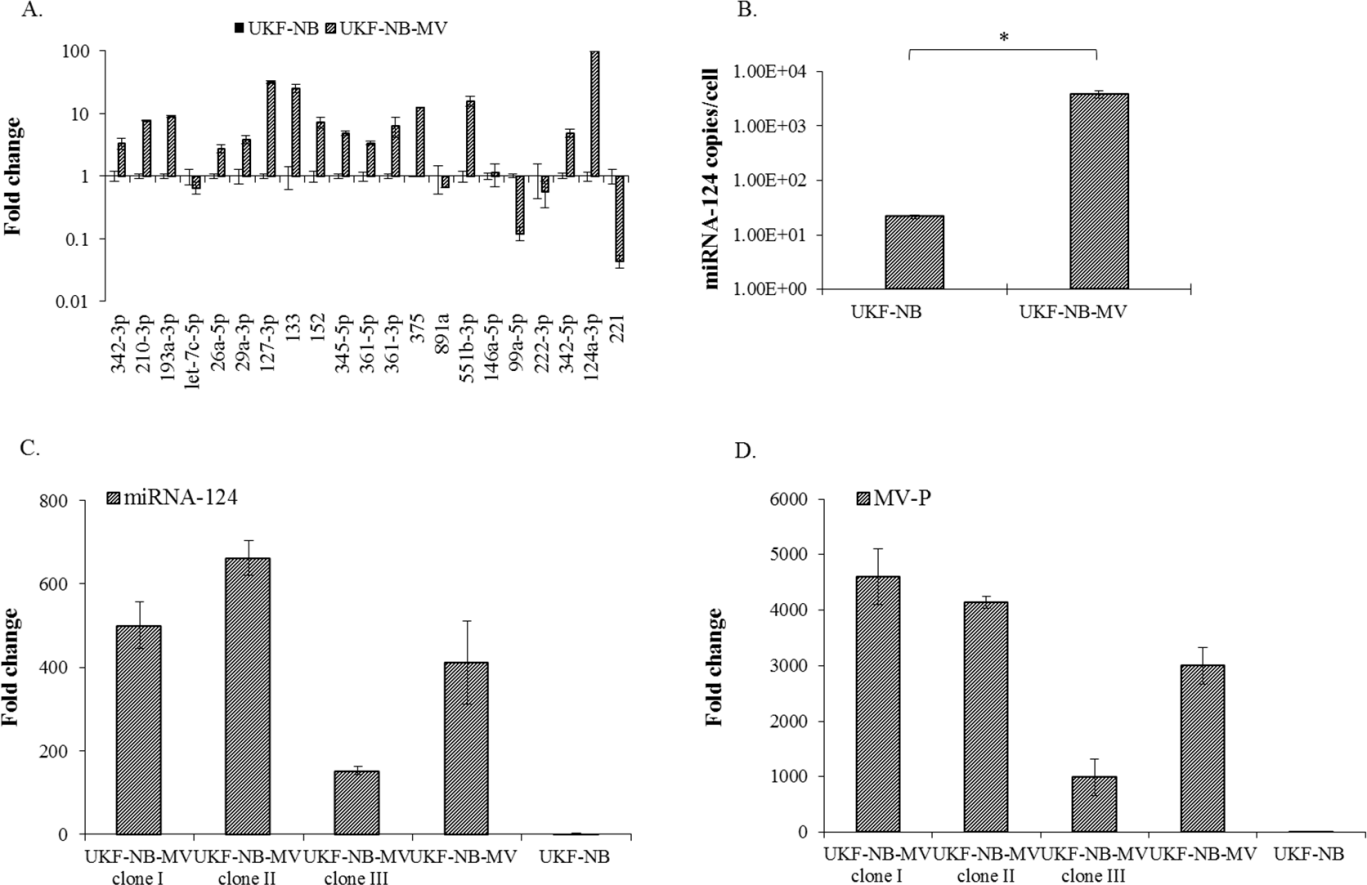
Differential expression of miRNAs in MV-persistently infected UKF-NB. (A) Expression levels of selected miRNAs between uninfected and MV persistently infected UKF-NB cells. The relative expression (fold change) of each miRNA was determined by qPCR and expressed as the fold change relative to the control U6 gene. Expression in the UKF-NB cell line was used as the reference between the infected and the uninfected cells. Three independent cDNA libraries of each cell line were tested three times, each time point in triplicate. Averages with SEs are indicated for three independent experiments. (B) To determine the absolute transcript copy number per cell of miRNA-124 in UKF-NB-MV and uninfected UKF-NB cells (2X10^5^/well), we used miRNA-124 qSTAR primer pairs and a commercial miRNA-124 copy number standard kit. Averages with SEs are indicated for three independent experiments each in triplicate. T—test was performed and significance of at least p<0.05 is shown (*). (C) Relative expression of miRNA-124 in different clones of UKF-NB-MV cells. cDNA libraries of three independent UKF-NB-MV clones (I, II, III) were prepared and miRNA-124 relative expression was determined by qPCR and normalized to U6. Expression in the uninfected UKF-NB cell line was used as the reference. Triplicate samples of three independent cDNA libraries of each clone were tested. Clonal variations were observed, averages with SEs are indicated. (D) Analysis of qPCR of MV-P RNA in the different clones, normalized to U6. Expression in the uninfected UKF-NB cell line was used as the reference. Triplicate samples of three independent cDNA libraries of each clone were tested. Differences between all the clones were observed. UKF-NB-MV (fourth column) represents the parental un-cloned cells. Averages with SEs are indicated.

To determine if host cell miRNAs expression contributes to MV viral persistence, we next compared expression levels of selected miRNAs between uninfected and persistently MV-infected UKF-NB cells, using real time PCR (qPCR) (Fig 2A) (see Materials and Methods). Of the many miRNAs that were affected, we focused on those that targeted genes involved in interferon synthesis and response. In addition, some of these miRNAs have an intriguing complementarity to MV genomic RNA or MV transcripts, suggesting a possible direct role of these differentially expressed miRNAs in viral replication. Although several miRNAs varied between the infected and non-infected cells, we chose to further investigate miRNA-124. This miRNA was expressed in the highest amounts in the persistently infected cells and is specifically related to cells from the neuronal linage. MiRNA-124 was strongly induced in the infected UKF-NB-MV cells, 100-fold by qPCR (Fig 2A). The number of copies / cell increased from 22±1.7 in the UKF-NB to 3.82X10^3^±5.4x10^2^ in UKF-NB-MV cells, a 176 fold increase (Fig 2B). Therefore, we further investigated this miRNA, especially as miRNA-124 is highly abundant in differentiated neuronal cells [14]. To confirm that our results were not due to artifacts of clonality, individual clones of UKF-NB-MV cells were isolated by limiting dilution. In Fig 2, we confirmed that three randomly chosen clones (Clone I, II and III) expressed various amounts of miRNA-124 (Fig 2C) and MV-P (Fig 2D). These results suggest that our observations are not due to artifacts of clonality but represent an average of the polyclonal UKF-NB-MV culture.

### Ectopic expression of miRNA-124 induces apoptosis in UKF-NB-MV cells, but not in UKF-NB cells

**Fig 3:**
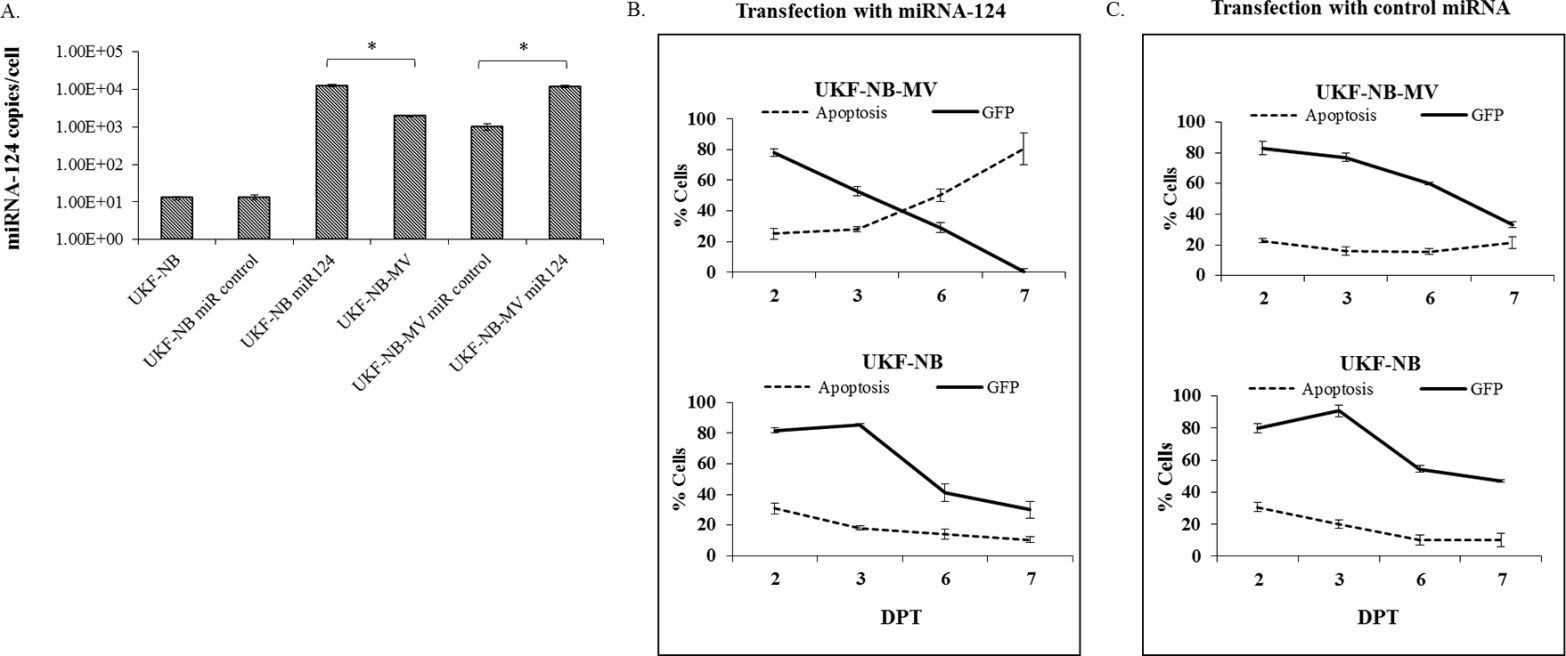
MiRNA-124 transfection induces apoptosis in UKF-NB-MV cells. To study the possible effects of ectopic overexpression of miRNA-124 on UKF-NB and UKF-NB-MV cells and apoptosis, the cells were first transfected with GFP-miRNA-124 (miR124) or empty GFP-plasmid (miR control). (A) Three days post transfection (DPT) we determined the absolute transcript copy number per cell of miRNA-124. Following treatment, RNA was extracted, cDNA libraries were prepared, and miRNA-124 concentrations were determined using qSTAR primer pairs and a commercial miRNA-124 copy number standard kit. Averages with SEs are indicated for three independent experiments each in triplicate. T-test was performed and significance of at least p<0.05 is shown (*). (B) Apoptosis with Annexin V and 7AAD staining (early and late apoptosis respectively, added as a single value) in UKF-NB-MV and UKF-NB cells was determined following transfection (DPT) with GFP-miRNA-124 (miR124) or (C) control plasmid-GFP (miR control). Apoptosis was determined by flow cytometry exclusively in the GFP positive populations. Averages with SEs of four similar experiments in triplicate are indicated.

To study the possible effects of ectopic overexpression of miRNA-124 on UKF-NB cells, UKF-NB and UKF-NB-MV cells were transiently transfected with a GFP-miRNA-124 plasmid. First, the presence of miRNA-124 in UKF-NB transfected cells was confirmed by qPCR, at three days after transfection. UKF-NB cells transfected with miRNA-124 showed equivalent amounts of miRNA-124 as similarly transfected UKF-NB-MV cells (Fig 3A). Following transfection, overall apoptosis was determined by FACS analysis (Fig 3B and 3C). Increased apoptosis (up to 80% on day 7) was observed only in GFP-positive UKF-NB-MV cells (Fig 3B). This effect was not observed in cells transfected with control plasmid or in uninfected UKF-NB cells, the extent of apoptosis in UKF-NB cells remained in the range of 15–30% over time, regardless of the transfected miRNA (Fig 3C). Thus, the extent of apoptosis was most likely due to the combination of both miRNA-124 and the presence of MV in these cells.

**Fig 4:**
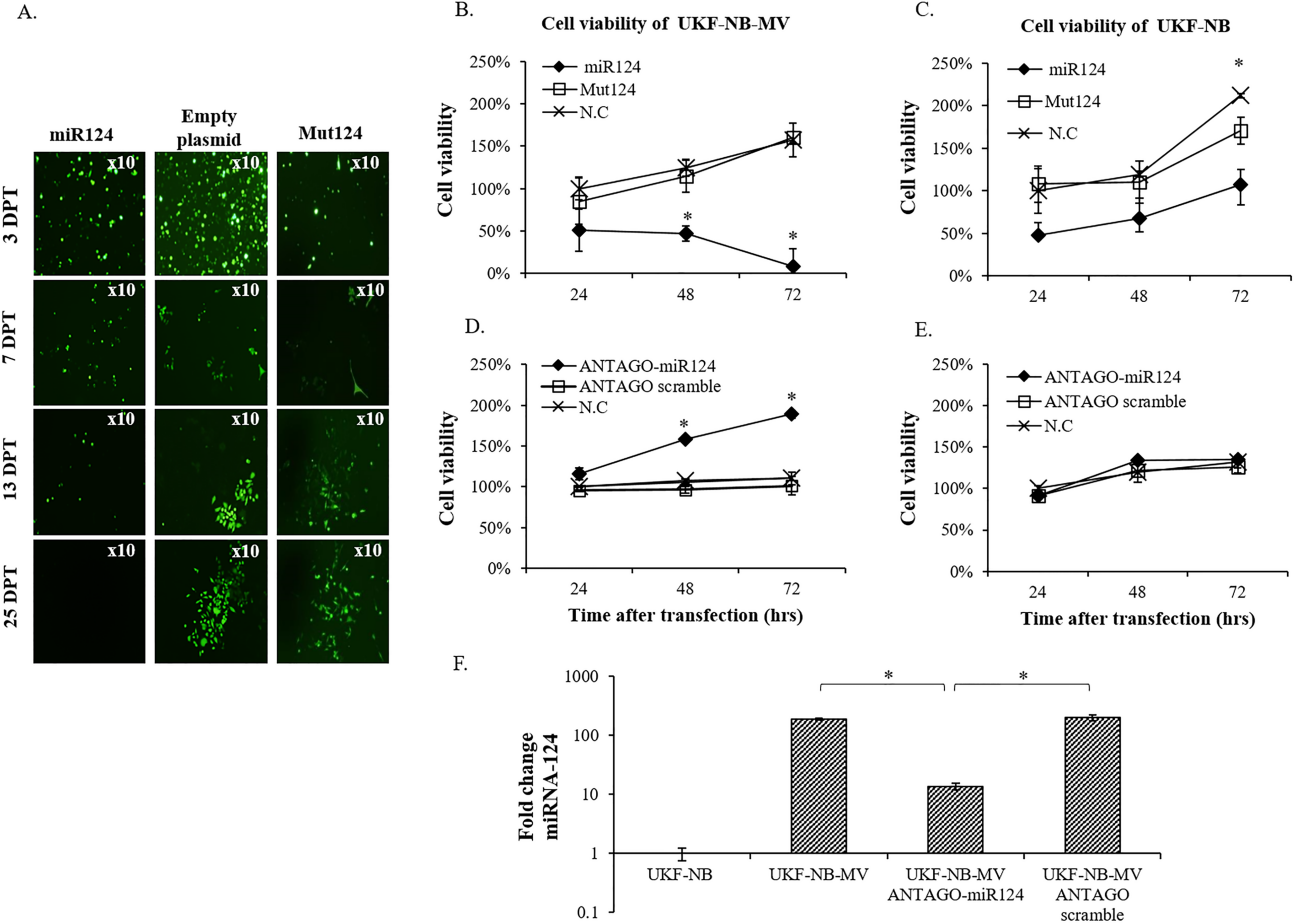
miRNA-124 overexpression induces cell death in UKF-NB-MV cells. (A) UKF-NB-MV cells were transfected with: GFP-miRNA-124 (miR124), empty GFP-plasmid, or GFP-MUT-miRNA-124 (Mut124). The transfected cells were sorted 3 days post transfection (DPT) and seeded into six well plates. GFP fluorescence of viable cells was followed for 25 days post transfection. The experiment was repeated at least three times. Efficiency of transfection with the three plasmids was >90%. Inhibition of miRNA-124 expression by transfection with ANTAGOmiRNA-124 induces increased cell proliferation in UKF-NB-MV cells, but not in UKF-NB cells. (B) UKF-NB-MV and (C) UKF-NB cells were transfected with GFP-miRNA-124 (miR124), GFP-MUT-miRNA-124(Mut124), or negative control (NC), empty plasmid-GFP. Cell viability was determined by XTT assay at different times post transfection (hrs). Values were expressed as a percentage compared to the negative controls. Significance between samples was determined by T-test, and p<0.05 between samples is shown (*). Efficiency of transfection with the three plasmids was >90%. The experiment was repeated three times. (D) UKF-NB-MV and (E) UKF-NB cells were transfected with ANTAGO miRNA-124 (ANTAGO-miR124), ANTAGO scrambled miRNA-124 (ANTAGO scramble) or mock transfection reagents (N.C). Cell viability was determined by XTT at different times post transfection (hrs). The experiment was repeated three times. Significance between samples was determined by T-test, and p<0.05 between samples is shown (*). Efficiency of transfection with the three plasmids was >90%. (F) miRNA-124 silencing efficiency was tested on persistently infected UKF-NB-MV and persistently infected cell that were transfected with ANTAGO miRNA-124 or ANTAGO scrambled miRNA-124. MiRNAs relative expression was determined at 72 hrs by qPCR and expressed as the fold change relative to U6. Expression in the UKF-NB cell line was used as the reference. Three independent cDNA libraries of each cell line were tested three times each. Averages with SEs are indicated. Significance between samples was determined by T-test, and p<0.05 between samples is shown (*).

To confirm these results further, we FACS-sorted the GFP positive cells three days after miRNA-124 transfection (Fig 4A). Ectopic overexpression of GFP-miRNA-124 in UKF-NB-MV caused overall cell death over time, similar to cisplatin treatment (Fig 1F). In contrast, UKF-NB-MV cells transfected with two different GFP control plasmids remained viable and proliferated. The loss of GFP-positive UKF-NB-MV cells transfected with miRNA-124 was not due to the loss of the plasmid.

To verify that these cells were dying, we determined cell viability by the XTT assay at three different times post-transfection (Fig 4B–4E). A significant decline in cell viability was observed exclusively in miRNA-124 over-expressing UKF-NB-MV cells (Fig 4B). UKF-NB cells overexpressing miRNA-124 had a slower growth rate than control UKF-NB cells (Fig 4C). Transfection of UKF-NB-MV cells with ANTAGOmiRNA-124, which inhibits miRNA-124 (from 186- to 13-fold change; Fig 4F), resulted in re-acquisition of rapid cell division, and reduced cell death (Fig 4D). Since UKF-NB cells express low basal levels of miRNA-124, transfection with ANTAGOmiRNA-124 did not change the cell's growth rate (Fig 4E). We conclude that overexpression of miRNA-124 is not sufficient to induce cell death, but can do so in the presence of persistent MV.

### Elevated miRNA-124 in MV persistently infected cells correlates with down-regulation of CDK6

**Fig 5:**
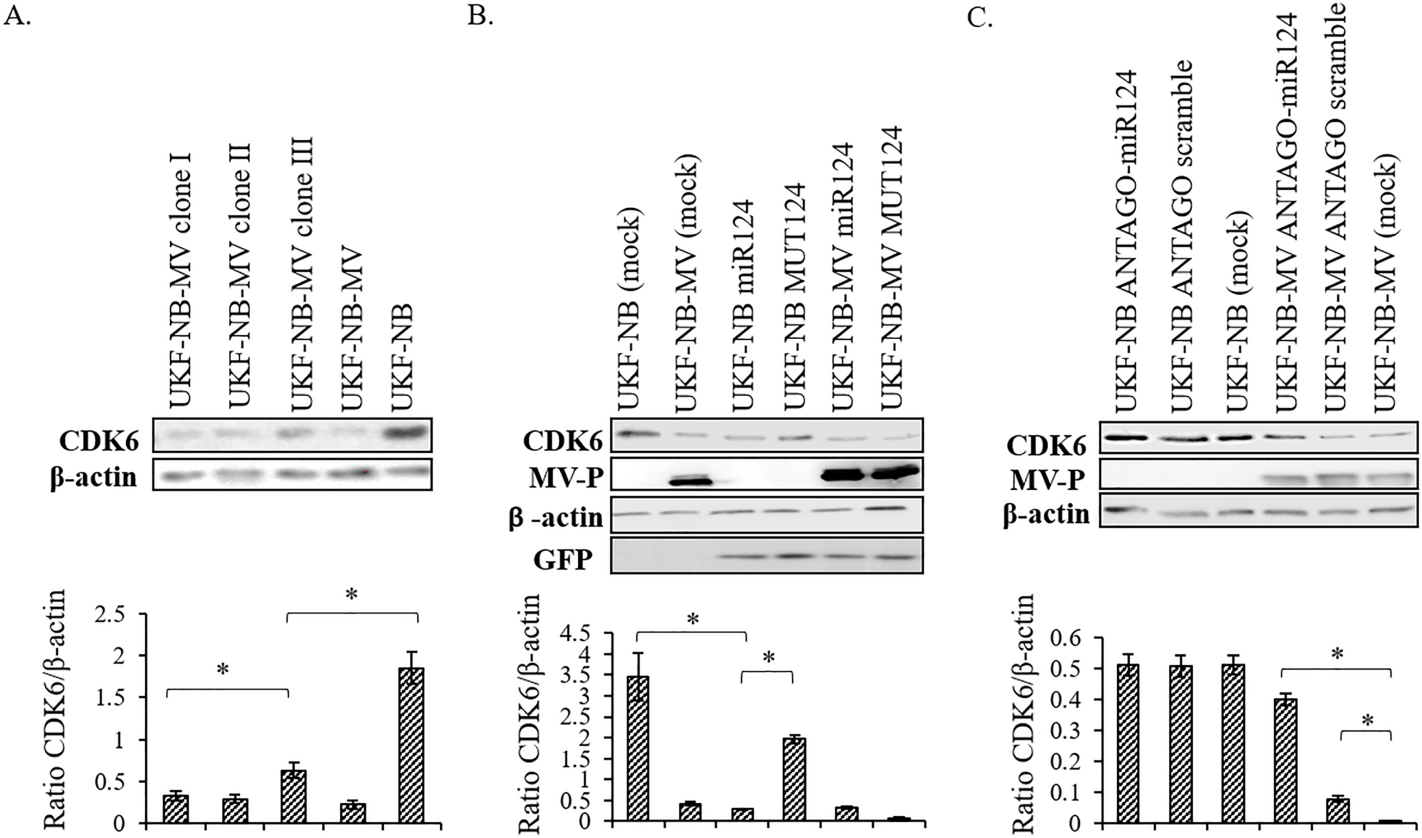
Downregulation of CDK6 following miRNA-124 expression. (A) We isolated single cell clones from the UKF-NB-MV cell culture by limiting dilution. We then determined the expression level of CDK6 at three independent UKF-NB-MV clones (I, II, III). CDK6 was detected by Western blot. A representative experiment out of three is presented. Experiments were repeated at least three times and quantified by densitometry. Densitometry averages with SEs of three bands obtained from three independent experiments are indicated. (B) UKF-NB or UKF-NB-MV cells were transfected with miRNA-124- GFP (miR124), or GFP-MUT-miRNA-124(Mut124). GFP fluorescent cells were determined to be 95% in both groups two days post transfection. Expression of CDK6, MV-P protein and β-actin was determined by Western blot. Cell lysates were prepared three days post transfection. A representative experiment out of five is presented. Experiments were repeated five times and quantified by densitometry (lower panel). Densitometry averages with SEs of the bands obtained from five independent experiments are indicated. Significance between samples was determined by T-test, and p<0.05 between samples is shown (*). (C) Transfection of UKF-NB or UKF-NB-MV cells with ANTAGO miRNA-124 (ANTAGO-miR124), scrambled ANTAGO miRNA-124 (ANTAGO scramble), or transfection reagents alone (mock). Expression of CDK6, MV-P protein and β-actin was determined by Western blot. Cell lysates were prepared three days post transfection. Experiments were repeated five times and quantified by densitometry analysis (lower panel). Densitometry averages with SEs of the bands obtained from five independent experiments are indicated. Significance between samples was determined by T-test, and p<0.05 between samples is shown (*).

Cyclin-dependent kinase 6 (CDK6) is an important regulator of cell cycle progression that enables the G1/S transition. CDK6 is also a known target gene for miRNA-124 [21], [29]. As miRNA-124 is strongly expressed in slowly dividing UKF-NB-MV cells, we first determined whether CDK6 was concomitantly down-regulated in individual clones of UKF-NB-MV cells isolated by limiting dilution (Fig 5A, Clone I, II and III). The clones expressed various amounts of miRNA-124 (Fig 2C), which inversely correlated with the amounts of CDK6 (Fig 5A).

Overexpression of miRNA-124 in uninfected UKF-NB cells also reduced CDK6 expression, implicating a direct effect of miRNA-124 on CDK6 expression levels. However, overexpression of miRNA-124 in UKF-NB-MV cells did not result in further reduction of CDK6. These cells express a very low amount of CDK6 to begin with, a level which is required for cell survival [18] (Fig 5B), and further reduction in CDK6 expression leads to cell death (see below).

Expression of ANTAGOmiRNA-124 prevented CDK6 down-regulation in UKF-NB-MV cells (Fig 5C). MiRNA-124 levels were determined 72 hrs. post-transfection with the ANTAGOmiRNA-124 (Fig 4F). A reduction in miRNA-124 levels correlated with increased cell proliferation. The presence of ANTAGOmiRNA-124 had no effect on UKF-NB cells, as these cells normally express low relative levels of basal miRNA-124 (Fig 5C). To further strengthen the correlation between CDK6 reduction and establishment of persistence, we silenced CDK6 expression using si-RNA. Unfortunately, following transfection, we were not able to recover viable cells (data not shown). These results, probably reflect the fact that neuroblastoma cells are uniquely sensitive to CDK6 inhibition [18]. Nonetheless these results are consistent with our observation that expression of miRNA-124 is associated with inhibition of CDK6 expression, accompanied by a concomitant decrease in cell proliferation.

**Fig 6:**
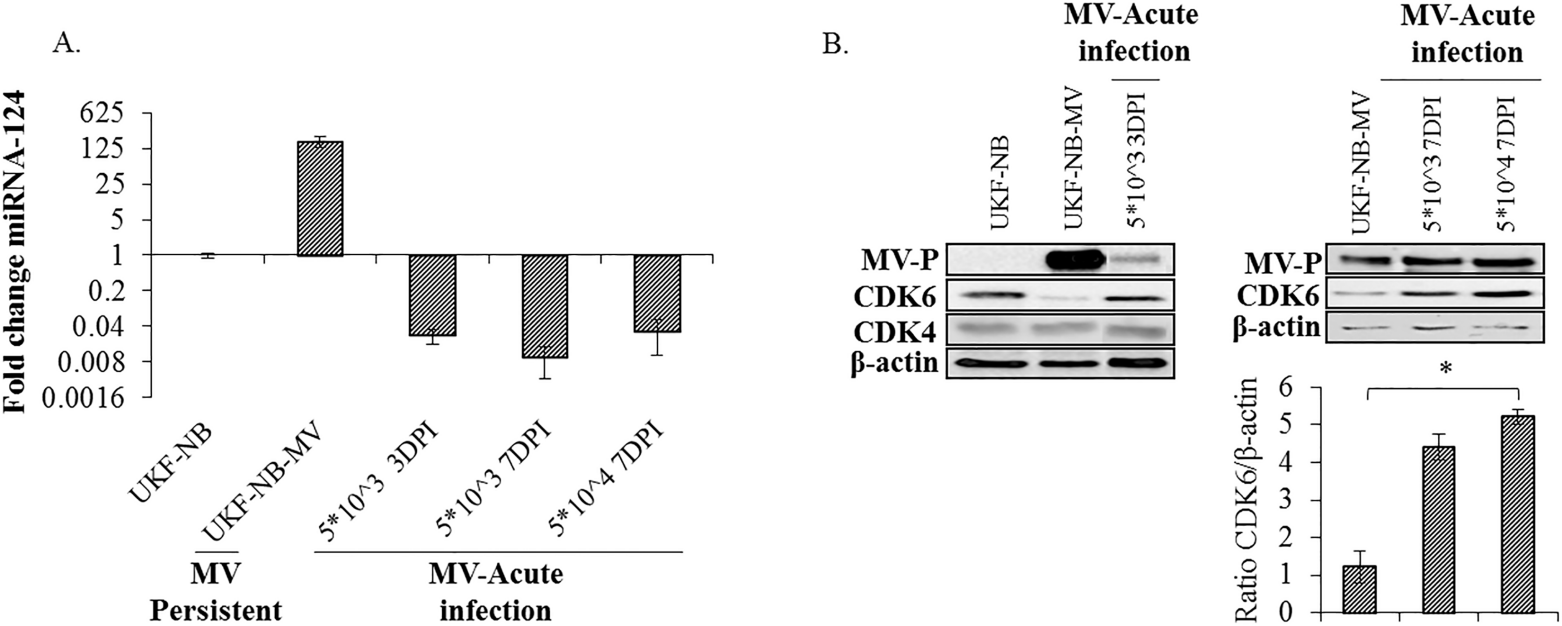
MiRNA-124 expression in MV acutely infected cells. (A) UKF-NB cells were acutely infected with the MV Edmonston strain at various doses, and then assessed at either 3 or 7 DPI. (5x10^3^ PFU/ml, MOI 0.005(3 DPI), 5x10^3^ PFU/ml (7 DPI) or 5x10^4^ PFU/ml, MOI 0.05 (7 DPI)). Three or seven DPI, cDNA libraries were prepared and miRNA-124 relative expression was determined by qPCR and expressed as the fold change relative to U6. Expression in the uninfected UKF-NB cell line was used as a negative control, and persistently infected UKF-NB-MV was used as a positive control. Three independent cDNA libraries were tested three times in triplicate. Averages with SEs are indicated for three independent experiments. (B) Three DPI, cell lysates were prepared. MiRNA-124 target protein CDK6 and negative control (CDK4) were detected by Western blot in uninfected (UKF-NB) cells, persistently infected (UKF-NB-MV) and acute infected UKF-NB cells (5x10^3^ PFU/ml 3DPI). Experiments were repeated at least three times and quantified by densitometry analysis. A representative experiment is presented. Densitometry averages with SEs of three bands obtained from three independent experiments are indicated. Significance between samples was determined by T-test, and p<0.05 between samples is shown (*).

Viral infection can affect the host cell gene expression enabling persistence. Alternatively a persistent infection could be established only in predefined, receptive sub-populations of cells. Thus, we tested whether MV infection up-regulates miRNA-124 expression in UKF cells. The results show that acute infection, as determined 3 and 7 DPI at two different virus inocula, neither increased the levels of miRNA-124 (Fig 6A) nor decreased CDK6 (Fig 6B), suggesting that the establishment of persistent infection in UKF-NB cells requires prior miRNA-124 expression, rather than its induction.

**Fig 7:**
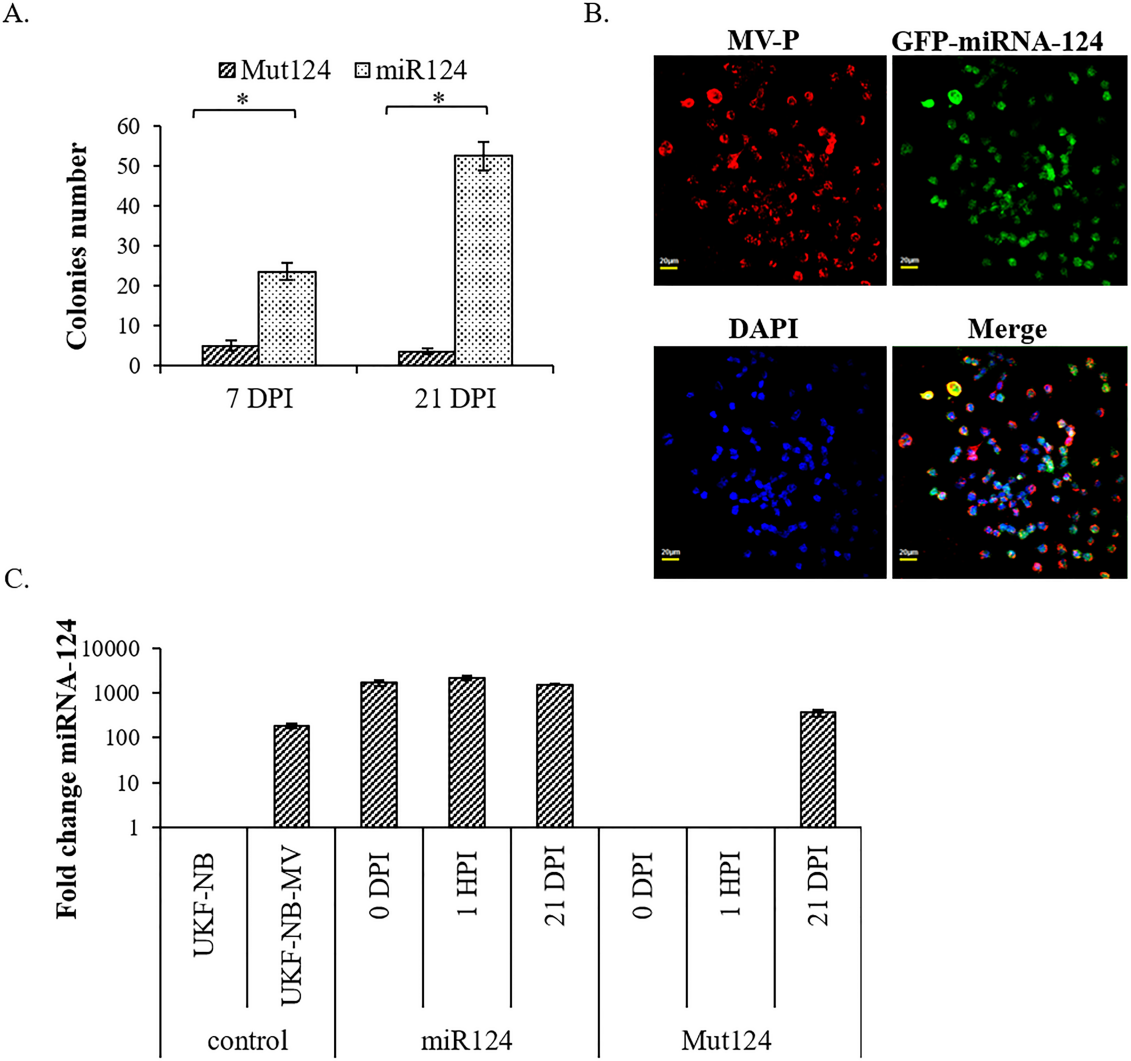
Stable miRNA-124 transfection of UKF-NB cells, followed by acute MV infection facilitated the establishment of persistently infected colonies. 2x10^4^ UKF-NB cells/well were seeded in triplicate. UKF-NB cells were stably transfected with constructs encoding GFP-miRNA-124 (miR124) or GFP-MUT-miRNA-124 (Mut124). Twenty days post transfection (DPT) GFP fluorescent cells were determined to be 95% in both groups. Cells monolayers were then acutely infected with Edmonston MV (5 x 10^3^ PFU /ml, MOI = 0.005). Most cells died, though surviving cells established colonies, which were scored. (A) Fixed and crystal-violet stained colonies were scored at day 7 and 21 post infection (DPI). The experiment was repeated three times. T-tests were performed and p<0.05 significance between samples is shown (*). (B) 21 DPI fluorescent staining of UKF-NB expressing GFP-miRNA-124(green), MV-P (red) and nuclear staining with DAPI (blue) is presented. Pictures were taken using confocal microscopy. All colonies were MV-positive and GFP-positive expressing the full length GFP-miRNA-124. (C) Establishment of MV persistent infection correlates with miRNA-124 expression. UKF-NB cells were seeded in triplicate. UKF-NB cells were stably transfected with constructs encoding GFP-miRNA-124(miR124) or the GFP-MUT-miRNA-124 (Mut124). Twenty days post transfection (DPT), GFP fluorescent cells were determined to be 95% in both groups. On day 21, cells monolayers were acutely infected with Edmonston MV. qPCR of miRNA-124 was determined before MV infection (0 DPI); 1 hour post infection (1 HPI) and 21 days post infection (DPI). The experiment was repeated three times for each independent infection. Averages with SEs are indicated. Significance between samples was determined by T-test, and p<0.05 between samples is shown (*).

To determine the contribution of miRNA-124 to the establishment of persistence we performed stable transfections of UKF-NB cells with GFP-miRNA-124 or GFP-mutant miRNA-124 (MUT- miRNA-124) (Fig 7). When all cells in both groups became GFP-positive, we acutely infected them with MV-Edmonston (5 x 10^3^ PFU /ml, MOI = 0.005). This MOI value was determined to be the lowest that induced infection but did not kill all cells in culture. Most of the cells died after infection as expected, but the few surviving cells formed colonies, which were scored 7 and 21 DPI (Fig 7A). At 21 DPI, there were 15-fold more GFP-miRNA-124 positive colonies as compared to control GFP-MUT-miRNA-124 cells. All persistently infected colonies were both GFP positive (transfected with either GFP-miRNA-124 or GFP-MUT-miRNA-124) and MV positive (Fig 7B).

MiRNA-124 was readily detected in pooled colonies after 21 DPI, suggesting that miRNA-124 expression is necessary for establishment of persistence (Fig 7C). Interestingly, UKF-NB cells transfected with MUT-miRNA-124 that survived acute infection with MV, and which were initially negative for miRNA-124, became positive after 21 days (Fig 7C). In addition the morphology of MV infected UKF-NB colonies resembled the morphology of UKF-NB-MV cells (Fig 1B), suggesting that MV persistence is associated with miRNA-124 expression and neuronal differentiation. We conclude that cells expressing miRNA-124, by either selection or transfection, become permissive to MV persistent infection.

## Discussion

While the field of miRNA's biology is expanding rapidly, few studies have explored the inter-relationships between host miRNAs expression and the life cycle of RNA viruses that themselves do not express miRNAs. The role of host miRNAs in the establishment of persistence by such a virus, the MV, has not yet been investigated. Therefore in this study, we described the contribution of miRNA-124 in persistent MV infection of neuroblastoma cells. MV does not encode miRNAs in its genome, is neurotropic, and, while cytopathic in certain non-neuronal cells, can persistently infect neurons and lead to severe CNS diseases in humans, including SSPE. In neuroblastoma cells supporting MV persistent infection, the expression of miRNA-124 was augmented. This result correlated with decreased CDK6 expression, a well-described target gene for miRNA-124, resulting in slower cell division. Ectopic overexpression of miRNA-124 led to a strong reduction in CDK6 protein levels and reduced the rate of cell proliferation. Stable transfection of UKF-NB cells with miRNA-124, followed by acute MV infection facilitated the establishment of persistently infected colonies.

MV is typically an acute, self-limited disease in which the virus appears to be eliminated, but may persist in the host CNS [30]. Possible mechanisms of persistence include the unique immune surveillance in the brain, accumulation of mutations in the viral genome [31–34] and unique attributes of CNS neurons that predispose to changes in the viral life cycle such as of virus assembly and budding [26]. SSPE is a rare and chronic neurodegenerative and neuro-inflammatory disease caused by MV persistent infection within the brain, in which neurons are the primary target. During SSPE, mature virions, containing antisense RNA, are rarely produced. In affected neurons in vitro, there is an accumulation of inclusion bodies containing nucleocapsids and surface proteins (H, F and M) as well as restricted release of infectious progeny and syncytia formation [35]. Within the limitations of this work, namely, one cell type and one MV strain, several similarities to persistent MV infection of neuronal cells in SSPE were observed. Persistently infected UKF-NB-MV divide slowly and produce large amounts of intracellular MV proteins, with limited production of infectious virions. To extend this findings further, beyond one cell line, to primary neuronal cells, we infected primary CD46+ hippocampal neurons from transgenic mice with MV [26]. In these cells, both the uninfected and persistently infected cells express high amounts of miRNA-124. Our preliminary data shows that MV persistent infection is readily attained in these cells without cytolysis. Thus, similarly to our results with UKF-NB-MV cells, the presence of miRNA-124 is associated with the ability of the virus to establish persistent infection.

Recently, Yis et al described the induction of three miRNAs (miRNAs 146a, 181a, and 155) in peripheral blood mononuclear cells of patients with SSPE, with no prognostic significance [36]. We examined these miRNAs in our neuroblastoma cells. Neither miRNA-146 nor miRNA-181a was detected. MiRNA-155 was increased two fold in MV infected cells (not shown); suggesting that specific miRNA expression in MV infection is cell type dependent. To our knowledge, with the exception of this report, the role of miRNAs in MV-related pathologies has not been investigated.

Several miRNAs vary between MV persistently infected and non-infected UKF-NB cells. While the contribution of additional miRNAs to MV persistent infection should be further investigated, we chose to focus on the contribution of a neuronal miRNA, miRNA-124, one of the best-characterized miRNAs in the CNS. MiRNA-124 level increases over time in the developing nervous system [14]. Furthermore, neuronal differentiation is enhanced following ectopic expression of miRNA-124 in mouse neuroblastoma cells [37], mouse embryonic carcinoma cells, and mouse embryonic stem cells [38] as well as neuronal differentiation of postnatal neural stem cells and glioma stem cells [39]. Cyclin dependent kinase 6 (CDK6) [21] and Solute Carrier Family 16 (SLC16A1) [40] are two identified key targets of miRNA-124 in Medulloblastoma, suggesting a role for this miRNA in cell proliferation and cell cycle. Pierson et al [21] have shown that miRNA-124 is down-regulated in medulloblastoma cells, and that its target, CDK6, is overexpressed [41]. CDK6 is an important regulator of cell cycle progression regulating the G1/S transition [19, 20]. MiRNA-124 plays a key role in cancer cell proliferation and is epigenetically silenced in various types of cancers [21, 39], and affects proliferation and motility of cancer cells by repressing ROCK2 and EZH2 [42]. We show that MV persistent infection of UKF-NB cells is accompanied by miRNA-124 increased expression, and reduced cell proliferation. Consistent with this finding, we also observed reduced CDK6 protein expression in UKF-NB-MV as compared to uninfected cells. It has been previously reported in peripheral blood lymphocytes that the MV induces G0/G1 cell cycle arrest, and also deregulates CDK/cyclin complexes essential for G1/S phase progression [16, 17].

Here, we provide evidence that expression of miRNA-124 in neuroblastoma cells is an important molecular link between MV persistent infection, CDK6 inhibition, and reduced cell division that facilitates establishment of persistent infection.

Several miRNA genes, including miRNA-124, harbor CpG islands that can undergo methylation-mediated silencing, a characteristic of many tumor suppressor genes. For example, miRNA-124 is frequently methylated in tumors [43]. Expression of miRNA-124 is therefore dependent on demethylation of its promoter. It would be of interest to determine whether the persistently infected UKF-NB-MV cells as well as the very small, uninfected UKF-NB cell subpopulation of miRNA-124 positive cells harbor demethylated miRNA-124 CpG islands. Stress by MV infection may also contribute to modulation of DNA methylation as shown by Zhou et al [44].

Host cell apoptosis is typically induced by acute MV infection [27, 44, 45]. Although the involvement of host miRNAs in apoptosis and viral infection has been reported for DNA viruses [46], little is known about how host miRNAs influence infections by RNA viruses. Recent evidence demonstrates that miRNAs can also affect RNA virus replication and pathogenesis through direct binding to the RNA virus genome or through virus-mediated changes in the host transcriptome [10]. In this report, we show that neuroblastoma cells persistently infected with MV had high levels of miRNA-124. These cells were viable, but divided more slowly and were more sensitive to death (both spontaneously and upon cisplatin treatment) when compared to uninfected cells (Fig 1D and 1F). These results seem to contradict the known role of miRNA-124 in protecting neurons from death [47]. Indeed, miRNA-124 overexpression promotes neuronal differentiation [14, 37] and reduces apoptosis in bone marrow-derived mesenchymal stem cells [48]. However, apoptosis is an important factor in host defense, limiting the replication and spread of viruses [49, 50]. As a consequence, many viruses have evolved various mechanisms to inhibit or evade apoptosis, often by affecting the expression of key cellular apoptotic proteins [51, 52]. With this in mind, we interpret our results as follows; MV infection of cells (including neuronal cells) triggers a cellular response that can result in apoptosis. In turn, MV infection can persist in cells that express miRNA-124, which plays a neuron-protective role, possibly through CDK6 reduction. Ectopic overexpression of miRNA-124 tilts this tenuous balance towards apoptosis and cell loss.

Low levels or absence of CDK6 is conducive to establishment of persistence that may also be attained in post-mitotic neuronal cells, since all CDKs (except of CDK5) in post-mitotic neurons are silenced [53]. Furthermore, post mitotic neuronal cells express high miRNA-124 levels [54]. Absence of CDK6 accompanied by high levels of miRNA-124 are consistent with the ability of MV to establish long-term persistent infection in non-dividing resident CNS cells [1]. In addition elevated miRNA-124 expression may promote neuronal differentiation of neuroblastoma cells, and the differentiated state is more conducive to a persistent infection. The fact that persistently infected cells consistently express elevated miRNA-124 may reflect viral selection of cells providing the appropriate, more differentiated environment.

In the context of miRNAs expression, two possible options for RNA virus persistence can be proposed: 1. The virus infects cells and induces changes in the host's miRNAs expression profile, allowing for persistent infection. Modulation of the miRNAs machinery by RNA viruses may confer multiple benefits for viral replication and survival in host cells. Supporting evidence for this mechanism can be found in several viruses: Yeung et al, showed changes in HeLa cellular miRNAs following HIV-1 infection [55]. Interestingly, HIV-1 decreased the expression of miRNAs-17/92, favoring its own replication [56]. By contrast, Chiang et al. reported that miRNA-132 enhances HIV-1 replication [57]. Hepatitis C virus (HCV) is another example of an RNA virus that modulates cellular miRNAs. MiRNAs may determine immune evasion and the rate of viral replication. HCV infection of hepatoma cells enhances miRNAs levels, which in turn inhibit an IFN-induced antiviral protein, and therefore aids in accelerated HCV replication and viral persistence [58]. In contrast to our results with the MV, Avila-Bonilla et al, recently reported the down regulation of miRNA-124 (among other miRNAs) in Dengue virus persistently infected versus acutely infected cells [59]. 2. The virus infects a cell type that expresses particular miRNAs, allowing the virus to persist. For instance, miRNA-122 is abundant in the liver and is essential to the stability and propagation of HCV RNA [60]. The miRNA-122–HCV complex protects the HCV genome from nucleolytic degradation and from host innate immune responses. The interaction of HCV with the liver-enriched miRNA, miRNA-122, represents an exceptional example of how RNA viruses can usurp host cellular machinery for their survival. HCV encodes for two binding sites in its 5′-UTR region for miRNA-122 [61]. Mechanistic studies have shown that binding of miRNA-122 to HCV RNA enhances RNA abundance, translation, and infectious virus production [62–64]. McQuaid et al found that MV can infect and replicate in undifferentiated and differentiated human neuronal cells [65]. To date, whether persistence occurs in particular neuronal subtypes, or at specific developmental stages is not known.

To further investigate the role of miRNA-124 in the establishment of persistence, uninfected cells were stably transfected with miRNA-124 followed by acute MV infection (Fig 7). The presence of miRNA-124 increases cell viability following MV infection, facilitating the establishment of persistent infection. These conclusions are strongly supported by the results in Fig 7C showing that stable UKF-NB MUT-miRNA-124 cells which survived acute infection and were initially miRNA-124 low expressers, were determined to be miRNA-124 positive after 21 days, presumably by selection of rare high threshold miRNA-124 positive cell subpopulations.

We are aware that MV related neuronal diseases are most likely due to wild type viral strains and not due to attenuated viral strains. However, we demonstrated the establishment of MV persistent infection in an additional unrelated cell and a wild type MV (BGU-iPSC cells kindly provided by Dr. Rivka Ofir) which was persistently infected with a GFP-labeled MV wild type strain, IC323-EGFP (MV-GFP; kindly provided by Dr. Yusuke Yanagi). BGU-iPSCs cells are pluripotent and can differentiate in vitro into germ layers including cells expressing the neuronal differentiation marker NF68. We determined that persistent infection with the wild type virus can be attained and miRNA-124 expression is significantly increased in the infected cells (data not shown).

Non-cytopathicity is a key element of persistent viruses. Surely miRNA-124 is not the sole cellular factor that predisposes to the establishment of MV persistence, but evidence presented here shows that selected miRNAs, which may be expressed only in particular cell populations, can influence the cell cycle and overall cell viability, both of which may be important in the establishment of persistent infections. Identification of novel cellular players may afford new opportunities to resolve or prevent debilitating chronic virus infections.

## Conclusions

Various factors allowing the MV virus to persist are known, yet, the role of miRNAs that favor establishment of a chronic, non-cytolytic infection by measles virus has not been studied. Here, we describe the contribution of miRNA-124 a host cell-encoded miRNAs in the development of MV persistent infection in the human neuroblastoma cell line UKF-NB (UKF-NB-MV). MiRNA-124 is strongly expressed in persistently infected cells and is abundant in normal neuronal cells. We propose that miRNA-124 limits CDK6 expression which in turn results in decreased cell proliferation, facilitating the establishment of MV persistence in neuroblastoma cells. Since, to our knowledge, this is the first report to describe the role of a specific miRNA in MV persistence.

## Materials and methods

### Cells and viruses

UKF human neuroblastoma cells (UKF-NB) were used in this study [25]. In addition, we used their MV persistently infected counterparts (UKF-NB-MV), developed following acute infection with the MV Edmonston vaccine strain (ATTC No VR-24). UKF-NB were kindly provided by Dr. Jindrich Cinatl, Jr., Dept. of Medical Virology, Goethe-University, Frankfurt, Germany. Aliquots of the cells were frozen. For experiments, both infected and non-infected cells were thawed and carried in parallel. Following primary infection (MOI = 0.005), cells in their 13 passage were used. The cell lines were grown in RPMI-1640 medium supplemented with 10% heat-inactivated fetal calf serum, 1% L-glutamine, and 1% pen-strep (Beit Haemek, Israel) and passaged by trypsinization.

### Antibodies

The following antibodies were used: MV Phosphoprotein (P) (ARGENE); MV matrix (M) (Abcam); CDK6 (Santa Cruz Biotechnology). CDK4 (Santa Cruz Biotechnology) is a functional homologue of CDK6 that is not a target for miRNA-124. Antibodies against it serve as a negative control in Western blots. CopGFP (Enzo Life Sciences & Axxora); GAPDH (MILLIPORE); β-actin (SIGMA-ALDRICH); anti-mouse and anti-rabbit IgG peroxidase Jackson ImmunoResearch Alexa flour®-488 conjugated goat anti-mouse and Alexa flour®-647 conjugated goat anti-rabbit (Molecular Probes Inc.). Fluorescence tags included Alexa Fluor 647-Annexin V, 7-AAD (BioLegend); DAPI (Sigma) and TO-PRO (ThermoFisher).

### Western blot analysis

Total cell lysates were extracted by RIPA lysis buffer containing 10 mM Tris pH 8.0, 100 mM NaCl, 5 mM EGTA, 0.1% SDS, 1% NP‑40, 45 mM β-mercaptoethanol, 50 mM NaF. Protease inhibitors (1 mM PMSF, 10 mg/ml aprotinine and 10 mg/ml leupeptin) were added immediately prior to cell lysis. Lysates were placed on ice for 30 min and sheared several times through a 21‑gauge needle, followed by centrifugation at 14,000 x g for 15 min at 4°C. Protein quantification of lysates was determined by the Bradford method (BioRad). Protein lysates (30 μg) were separated in 10% SDS-polyacrylamide gel and blotted onto nitrocellulose membranes. The membranes were incubated with several primary antibodies followed by anti-mouse or anti-rabbit peroxide-linked IgG. Protein bands were detected by chemiluminescence with ECL (Amersham).

### RNA extraction

Total RNA was extracted 3 days post infection using EZ-RNA II isolation kit (Biological Industries, Beit Haemek, Israel), according to the manufacturer’s protocol. Aliquots of total RNA were used directly for miRNAs quantification using qPCR or subjected to small RNA library construction.

### qPCR analysis of miRNAs in different cell lines infected and uninfected with MV

The miRNAs chosen for analysis were determined as follows: host miRNAs expression profiles were compared between three different MV persistently infected cells and their uninfected counterparts (two Hodgkin's lymphoma lines and one neuroblastoma cell line) (unpublished data). An integrated bioinformatic platform, developed previously in collaboration with Rosetta Genomics (Israel) was used. This platform is based on a custom-made microarray (MIRCHIP, Agilent) on which probes for all known annotated human and viral miRNAs, as well as bioinformatically predicted host- and virally-encoded miRNAs, are arrayed. Total RNA derived from the six cell populations (infected and uninfected) was applied to the arrays, and the unbiased presence of candidate miRNAs were discerned by differential light emission. Of the many miRNAs that were affected, we focused on those that targeted genes involved in interferon synthesis and response. In addition, some of these miRNAs have an intriguing homology to MV genomic RNA or MV transcripts, suggesting a possible direct role of these differentially expressed miRNAs in viral replication. For validation, cDNA libraries were prepared from UKF-NB and UKF-NB-MV cells using miScript Reverse Transcription Kit (QIAGEN, Hilden, Germany). Briefly, 1 μg of total RNA was poly-adenylated by poly (A) polymerase and converted into cDNA by reverse transcriptase with oligo-dT in a single step, at 37^o^ C for 1 hr. and 95^o^ C for 5 min. The cDNA libraries were used in the qPCR analysis for miRNAs using miScript SYBR Green PCR Kit (QIAGEN, Hilden, Germany) with a forward specific miRNA primer (sequences were taken from the DS analysis, Metabion Martinsried, Germany) and the miScript Universal reverse primer. Forward primers with high G/C content at the 3' end, were extended with one or two 'A' nt at their 3'-end. qPCR reactions were performed using Light Cycler 480 system (Roche Applied Science, Mannheim, Germany). The sequences of the reference genes are as follows:

miRNA hsa -124a-3p (forward primer: 5'- TAAGGCACGCGGTGAATGCC -3').
miRNA hsa-342-3p (forward primer: 5'-TCTCACACAGAAATCGCACCCG-3').
miRNA hsa-210-3p (forward primer:5'- CTGTGCGTGTGACAGCGGCTGA-3').
miRNA hsa-193a-3p (forward primer: 5'- AACTGGCCTACAAAGTCCCA-3').
miRNAhsa-let-7c-5p(forward primer:5'-TGAGGTAGTAGGTTGTATGGTT3').
miRNA hsa-26a-5p (forward primer: 5'- TTCAAGTAATCCAGGATAGGCT-3').
miRNA hsa-29a-3p (forward primer: 5'- TAGCACCATCTGAAATCGGT-3').
miRNA hsa-127-3p (forward primer: 5'- TCGGATCCGTCTGAGCTTGG-3').
miRNA hsa-133 (forward primer: 5'- TTTGGTCCCCTTCAACCAGCT-3').
miRNA hsa-152 (forward primer: 5'- TCAGTGCATGACAGAACTTGG-3').
miRNA hsa-345-5p (forward primer: 5'- GCTGACTCCTAGTCCAGGGCT-3').
miRNA hsa-361-5p (forward primer: 5'- TTATCAGAATCTCCAGGGGT-3').
miRNA hsa-361-3p (forward primer: 5'- TCCCCCAGGTGTGATTCTGAT-3').
miRNA hsa-375 (forward primer: 5'- TTTGTTCGTTCGGCTCGCGT-3').
miRNA hsa-891a (forward primer: 5'- TGCAACGAACCTGAGCCACT-3').
miRNA hsa-551b-3p (forward primer: 5'- GCGACCCATACTTGGTTTCA-3').
miRNA hsa-146a-5p (forward primer: 5'- TGAGAACTGAATTCCATGGGT-3').
miRNA hsa-99a-5p (forward primer: 5'- AACCCGTAGATCCGATCTTGT-3').
miRNA hsa -222-3p (forward primer: 5'- AGCTACATCTGGCTACTGGGT-3').

To determine the absolute transcript copy number of miRNA-124, 2X10^5^cells were seeded in 6-well plates. Following treatment, RNA was extracted, cDNA libraries were prepared (see above), and miRNA-124 copy numbers per cell were determined using qSTAR primer pairs and a commercial miRNA-124 copy number standard kit (ORIGENE).

### Evaluation of apoptosis by FACS analysis

For quantitative analysis of early apoptosis, Annexin V staining was performed. Briefly, 1 x 10^6^ cells were collected by centrifugation. The cells were resuspended in 1ml of binding buffer (BioLegend) and incubated for 15 min at room temperature in the dark with 10 μl of 647 Annexin V and 5 μl of 7-AAD for late apoptosis. Both early and late apoptosis values were added and presented as a single apoptosis value. Finally, cells were suspended in 400 μl of binding buffer and then analyzed by flow cytometry with a FACScan flow cytometer (Becton Dickinson) using CellQuest software.

### Cell sorting of purified cell subpopulations

GFP positive and negative UKF-NB-MV transfected cells were sorted sterilely with a SY3200 Cell Sorter (Sony Biotechnology, Inc.) and collected for further growth. We obtained two subpopulations: GFP positive cells transfected with GFP-miRNA-124 or GFP-control plasmids (see below). Single cell suspensions were prepared (2 x 10^6^ cells in 500 µl of sterile PBS). A GFP negative control (mock-transfected UKF-NB-MV) was used to detect auto-fluorescence. The cells were collected into BD Falcon 12x75 mm polystyrene tubes coated with 4% BSA. The sorting media was RPMI-1640 supplemented with 10% heat-inactivated fetal calf serum, 1% L-glutamine, and 1% pen-strep). Cells were sorted two days after transfection where >90% of the cells were GFP positive. 30,000 cells/well of each type were collected and plated in 6-well plates.

### Transfections

Hsa 124a-3p DNA constructs. We generated expression constructs in (pGreenPuro™ shRNA Cloning and Expression Lentivector, SBI) of both hsa -124a-3p and a 3' seed mutant by cloning the BamHI to EcoRI genomic fragment under the control of the H1 RNA polymerase III promoter and copepod green fluorescent protein (CopGFP) under the human cytomegalovirus (CMV) constitutive promoter. The sequences of the fragments are as follows:

Hsa-124a-3p:

5'GATCCT**AAGGC**ACGCGGTGAATGCCCTTCCTGTCAGAGGCATTCACCGCGT**GCCTT**ATTTTTG -3'
5'AATTCAAAAAT**AAGGC**ACGCGGTGAATGCCTCTGACAGGAAGGGCATTCACCGCGT**GCCTTA**G -3'

Mutant hsa-124a-3p (MUT-miRNA-124):

5'GATCCT**TTCCG**ACGCGGTGAATTCCCTTCCTGTCAGAGGAATTCACCGCGT**CGGAA**ATTTTTG -3'
5'AATTCAAAAAT**TTCCG**ACGCGGTGAATTCCTCTGACAGGAAGGGAATTCACCGCGT**CGGAAA**G -3'

Cells were transfected with the plasmid containing the miRNA-124, the mutant MUT-miRNA-124 or with the control plasmid lacking the insert. Briefly, transfection was performed using Lipofectamine™ 2000 Transfection Reagent. Cells (1x10^6^) were suspended in 1 ml of RPMI-1640 medium with FCS but without antibiotics, to a well of a 6-well plate 1 day before the transfection. For each well, we diluted 1.5 μg of DNA into 100 μl of medium without serum (Opti-MEM^®^). For each well, we diluted 4.5 μl of Lipofectamine™ 2000 into 100 μl OptiMEM I Medium and incubated for 5 min at room temperature and the DNA was added for 20 min. The solution was transferred directly to the cells. The plate was gently rocked and further incubated for 4 h at 37^o^ C in a CO_2_ incubator. Stable transfection with the same plasmids was performed as described above. Stable transfectants (UKF-NB miRNA-124 or MUT-miRNA-124) were established following selection with 1μg/ml puromycin.

Transfection with the miRNA-124 antisense. Cells were transfected with the inhibitor miRNA-124 antisense (Ambion). Briefly, transfection was performed using Lipofectamine™ RNAiMAX Transfection Reagent. Cells (1x10^6^) were suspended with 1 ml of growth medium with serum but without antibiotics, to a well of a 6-well plate (RPMI‑1640 medium, with L‑glutamine, containing 10% FCS) 1 day before the transfection. For each well, we diluted 30 pmol of DNA into 150 μl of medium without serum (Opti-MEM). For each well, we diluted 9 μl of Lipofectamine™ RNAiMAX into 150 μl OptiMEM® I Medium. Once the Lipofectamine™ 2000 was diluted, it was combined (1:1 ratio) with the DNA for 5 min. The DNA-Lipofectamine™ RNAiMAX complex was added directly to each well containing cells. The plate was mixed gently by rocking back and forth. The plate was incubated for 3 days at 37^o^ C in a CO_2_ incubator.

### Immunofluorescence staining

2.5x10^4^ cells were grown on glass slides (18x18 mm) and placed in wells of a 6-well plate with 2 ml growth media overnight. Cells were fixed in 4% paraformaldehyde for 20 min at room temperature. The cells were washed once with PBS, and kept in cold PBS until staining. Cells were incubated in blocking solution, 5% FCS, 0.5% Triton X-100, in PBS, for 30 min at room temperature. The cells were then incubated with the primary antibody for 2 hr at room temperature in humidified chamber. Cells were washed 3 times with PBS and then incubated with the fluorescent secondary antibody for 1 hr in the same conditions, protected from light. Cells were washed 3 times with PBS and stained with the nuclear dyes DAPI or TO-PRO for 15 min. Mounting medium (DAKO Cytomation) and a cover glass were added. The primary antibodies used were anti-P and F. For florescence detection of P, the secondary antibody was Alexa Fluor®-488 conjugated goat anti-mouse (green) and for F, Alexa Fluor®-647 conjugated goat anti-rabbit (red). Slides were kept in the dark at 4^o^ C until used. The cells were photographed with an Olympus Fluoview-FV-1000 confocal microscope.

### Cell growth

2x10^4^ cells/well were seeded in 6-well plates and incubated for several days. The numbers of cells, as well as the percentage of dead cells, were determined each day by the ADAM—Automatic fluorescence cell counter and the ADAM-MC kit.

### XTT-cell viability assay

Cell viability was measured by a tetrazolium-formazan XTT assay kit (Beit Haemek, Israel) in 96-well plates. 3×10^4^ cells/well in 100 μl medium were treated with different concentrations of cisplatin (Pharmachemie B.V., Holland) for 48 hr at 37°C. XTT solution (25 μl) was added, and the plates were again incubated for 4 hr at 37°C. Absorbance was read at 450 nm by an ELISA reader. Similarly, this assay was also used to determine cell-viability following transfections as described above.

### Preparation of cells for electron microscopy

Cells were washed in PBS three times for 5 min, fixed as a monolayer in 0.2% gluteraldehyde in 0.05 M cacodylate buffer (pH 7.2) for 3 min at room temperature and harvested with a plastic scraper. The collected cells were fixed again with 2% gluteraldehyde in 0.05 M cacodylate buffer at 4°C for 60 min. After 3 washes in 0.05 M cacodylate buffer the cell pellet was post-fixed in 1% OsO_4_ in cacodylate buffer at 4°C for 60 min. The cells were washed twice for 10 min in cacodylate buffer and dehydrated in graded ethanol concentrations, and embedded in Araldite mixture. Thin sections were stained in uranyl acetate for 15 min and observed in a transmission Electron Microscope-JEM 2100F.

### Measles virus infection

Acute infection of UKF-NB cells with the MV Edmonston strain: 1x10^6^ cells/well UKF-NB, UKF-NB miRNA-124 or MUT-miRNA-124 transfected cells were seeded in a 6-well plate in 2 ml growth media overnight. Cells were infected with 5x10^3^ PFU/ml (MOI = 0.005) for 60 min at 37^o^ C the plate were shaken every 15 min. The cells were then washed three times with PBS. Two ml of growth media was added to each well and grown for several days. The samples were tested by Western blot and qPCR.

### Clonal isolation by limiting dilution

UKF-NB-MV cells (0.1 ml) were seeded at a density of 10 cells/0.1 ml per well, in a 96-well tissue culture plate. The number of cells per well after 18–24 hours was assess microscopically. The wells with only one cell were noted and on the 4th day, colonies were assessed. The clones were grown to confluence and transferred to 24 well plates for expansion. Three representative clones were further analyzed by Western blot and qPCR.

## References

[1] Griffin DE, Lin WH, Pan CH. Measles virus, immune control, and persistence. FEMS Microbiol Rev. 2012;36(3):649–62. doi: 10.1111/j.1574-6976.2012.00330.x . 22316382 10.1111/j.1574-6976.2012.00330.x PMC3319515

[2] Moss WJ, Griffin DE. Measles Lancet. 379 England: 2012 Elsevier Ltd; 2012 p. 153–64. doi: 10.1016/S0140-6736(10)62352-5 21855993 10.1016/S0140-6736(10)62352-5

[3] Lin WH, Kouyos RD, Adams RJ, Grenfell BT, Griffin DE. Prolonged persistence of measles virus RNA is characteristic of primary infection dynamics. Proc Natl Acad Sci U S A. 2012;109(37):14989–94. doi: 10.1073/pnas.1211138109 . 22872860 10.1073/pnas.1211138109 PMC3443140

[4] Schneider-Schaulies J, Niewiesk S, Schneider-Schaulies S, ter Meulen V. Measles virus in the CNS: the role of viral and host factors for the establishment and maintenance of a persistent infection. J Neurovirol. 1999;5(6):613–22. Epub 1999/12/22. . 10602402 10.3109/13550289909021290

[5] Rall GF. Measles virus 1998–2002: progress and controversy. Annu Rev Microbiol. 2003;57:343–67. Epub 2003/10/07. doi: 10.1146/annurev.micro.57.030502.090843 . 14527283 10.1146/annurev.micro.57.030502.090843

[6] Rima BK, Duprex WP. Molecular mechanisms of measles virus persistence. Virus Res. 111 Netherlands2005 p. 132–47. doi: 10.1016/j.virusres.2005.04.005 15893837 10.1016/j.virusres.2005.04.005

[7] Doi T, Kwon HJ, Honda T, Sato H, Yoneda M, Kai C. Measles virus induces persistent infection by autoregulation of viral replication. Sci Rep. 2016;6:37163 doi: 10.1038/srep37163 . 27883010 10.1038/srep37163 PMC5121633

[8] Bartel DP. MicroRNAs: genomics, biogenesis, mechanism, and function. Cell. 116 United States2004 p. 281–97. 14744438 10.1016/s0092-8674(04)00045-5

[9] Cullen BR. Transcription and processing of human microRNA precursors. Mol Cell. 16 United States2004 p. 861–5. doi: 10.1016/j.molcel.2004.12.002 15610730 10.1016/j.molcel.2004.12.002

[10] Trobaugh DW, Klimstra WB. MicroRNA Regulation of RNA Virus Replication and Pathogenesis. Trends Mol Med. 23 England: 2016 Elsevier Ltd; 2017 p. 80–93. doi: 10.1016/j.molmed.2016.11.003 27989642 10.1016/j.molmed.2016.11.003 PMC5836316

[11] Bushati N, Cohen SM. microRNA functions. Annu Rev Cell Dev Biol. 2007;23:175–205. Epub 2007/05/18. doi: 10.1146/annurev.cellbio.23.090506.123406 . 17506695 10.1146/annurev.cellbio.23.090506.123406

[12] Lewis BP, Burge CB, Bartel DP. Conserved seed pairing, often flanked by adenosines, indicates that thousands of human genes are microRNA targets. Cell. 120 United States2005 p. 15–20. doi: 10.1016/j.cell.2004.12.035 15652477 10.1016/j.cell.2004.12.035

[13] Gottwein E, Cullen BR. Viral and cellular microRNAs as determinants of viral pathogenesis and immunity. Cell Host Microbe. 2008;3(6):375–87. doi: 10.1016/j.chom.2008.05.002 . 18541214 10.1016/j.chom.2008.05.002 PMC3079432

[14] Smirnova L, Grafe A, Seiler A, Schumacher S, Nitsch R, Wulczyn FG. Regulation of miRNA expression during neural cell specification. Eur J Neurosci. 21 France2005 p. 1469–77. doi: 10.1111/j.1460-9568.2005.03978.x 15845075 10.1111/j.1460-9568.2005.03978.x

[15] Lagos-Quintana M, Rauhut R, Yalcin A, Meyer J, Lendeckel W, Tuschl T. Identification of tissue-specific microRNAs from mouse. Curr Biol. 12 England2002 p. 735–9. 12007417 10.1016/s0960-9822(02)00809-6

[16] Engelking O, Fedorov LM, Lilischkis R, ter Meulen V, Schneider-Schaulies S. Measles virus-induced immunosuppression in vitro is associated with deregulation of G1 cell cycle control proteins. J Gen Virol. 1999;80 (Pt 7):1599–608. Epub 1999/07/28. doi: 10.1099/0022-1317-80-7-1599 . 10423127 10.1099/0022-1317-80-7-1599

[17] Naniche D, Reed SI, Oldstone MB. Cell cycle arrest during measles virus infection: a G0-like block leads to suppression of retinoblastoma protein expression. J Virol. 1999;73(3):1894–901. . 9971768 10.1128/jvi.73.3.1894-1901.1999 PMC104430

[18] Rader J, Russell MR, Hart LS, Nakazawa MS, Belcastro LT, Martinez D, et al Dual CDK4/CDK6 inhibition induces cell-cycle arrest and senescence in neuroblastoma. Clin Cancer Res. 2013;19(22):6173–82. doi: 10.1158/1078-0432.CCR-13-1675 . 24045179 10.1158/1078-0432.CCR-13-1675 PMC3844928

[19] Malumbres M, Barbacid M. Cell cycle, CDKs and cancer: a changing paradigm. Nat Rev Cancer. 9 England2009 p. 153–66. doi: 10.1038/nrc2602 19238148 10.1038/nrc2602

[20] Musgrove EA, Caldon CE, Barraclough J, Stone A, Sutherland RL. Cyclin D as a therapeutic target in cancer. Nat Rev Cancer. 11 England2011 p. 558–72. doi: 10.1038/nrc3090 21734724 10.1038/nrc3090

[21] Pierson J, Hostager B, Fan R, Vibhakar R. Regulation of cyclin dependent kinase 6 by microRNA 124 in medulloblastoma. J Neurooncol. 2008;90(1):1–7. Epub 2008/07/09. doi: 10.1007/s11060-008-9624-3 . 18607543 10.1007/s11060-008-9624-3

[22] Silber J, Hashizume R, Felix T, Hariono S, Yu M, Berger MS, et al Expression of miR-124 inhibits growth of medulloblastoma cells. Neuro Oncol. 2013;15(1):83–90. doi: 10.1093/neuonc/nos281 . 23172372 10.1093/neuonc/nos281 PMC3534424

[23] Ponomarev ED, Veremeyko T, Barteneva NS. Visualization and quantitation of the expression of microRNAs and their target genes in neuroblastoma single cells using imaging cytometry. BMC Res Notes. 2011;4:517 doi: 10.1186/1756-0500-4-517 . 22123030 10.1186/1756-0500-4-517 PMC3250958

[24] Wong KY, So CC, Loong F, Chung LP, Lam WW, Liang R, et al Epigenetic inactivation of the miR-124-1 in haematological malignancies. PLoS One. 2011;6(4):e19027 doi: 10.1371/journal.pone.0019027 . 21544199 10.1371/journal.pone.0019027 PMC3081325

[25] Cinatl J Jr., Cinatl J, Vogel JU, Kotchetkov R, Driever PH, Kabickova H, et al Persistent human cytomegalovirus infection induces drug resistance and alteration of programmed cell death in human neuroblastoma cells. Cancer Res. 1998;58(2):367–72. Epub 1998/01/27. . 9443419

[26] Lawrence DM, Patterson CE, Gales TL, D'Orazio JL, Vaughn MM, Rall GF. Measles virus spread between neurons requires cell contact but not CD46 expression, syncytium formation, or extracellular virus production. J Virol. 2000;74(4):1908–18. . 10644364 10.1128/jvi.74.4.1908-1918.2000 PMC111669

[27] Esolen LM, Park SW, Hardwick JM, Griffin DE. Apoptosis as a cause of death in measles virus-infected cells. J Virol. 1995;69(6):3955–8. . 7745753 10.1128/jvi.69.6.3955-3958.1995 PMC189125

[28] McQuaid S, McMahon J, Herron B, Cosby SL. Apoptosis in measles virus-infected human central nervous system tissues. Neuropathol Appl Neurobiol. 1997;23(3):218–24. Epub 1997/06/01. . 9223131

[29] Sherr CJ, Roberts JM. Inhibitors of mammalian G1 cyclin-dependent kinases. Genes Dev. 1995;9(10):1149–63. Epub 1995/05/15. . 7758941 10.1101/gad.9.10.1149

[30] Katayama Y, Hotta H, Nishimura A, Tatsuno Y, Homma M. Detection of measles virus nucleoprotein mRNA in autopsied brain tissues. J Gen Virol. 1995;76 (Pt 12):3201–4. Epub 1995/12/01. doi: 10.1099/0022-1317-76-12-3201 . 8847530 10.1099/0022-1317-76-12-3201

[31] Baczko K, Liebert UG, Billeter M, Cattaneo R, Budka H, ter Meulen V. Expression of defective measles virus genes in brain tissues of patients with subacute sclerosing panencephalitis. J Virol. 1986;59(2):472–8. . 3735490 10.1128/jvi.59.2.472-478.1986 PMC253098

[32] Cattaneo R, Rose JK. Cell fusion by the envelope glycoproteins of persistent measles viruses which caused lethal human brain disease. J Virol. 1993;67(3):1493–502. . 8437226 10.1128/jvi.67.3.1493-1502.1993 PMC237519

[33] Cattaneo R, Schmid A, Eschle D, Baczko K, ter Meulen V, Billeter MA. Biased hypermutation and other genetic changes in defective measles viruses in human brain infections. Cell. 55 United States1988 p. 255–65. 3167982 10.1016/0092-8674(88)90048-7 PMC7126660

[34] Watanabe M, Wang A, Sheng J, Gombart AF, Ayata M, Ueda S, et al Delayed activation of altered fusion glycoprotein in a chronic measles virus variant that causes subacute sclerosing panencephalitis. J Neurovirol. 1995;1(5–6):412–23. Epub 1995/12/01. . 9222385 10.3109/13550289509111034

[35] Patterson JB, Cornu TI, Redwine J, Dales S, Lewicki H, Holz A, et al Evidence that the hypermutated M protein of a subacute sclerosing panencephalitis measles virus actively contributes to the chronic progressive CNS disease. Virology. 291 United States: (C)2001 Elsevier Science.; 2001 p. 215–25. doi: 10.1006/viro.2001.1182 11878891 10.1006/viro.2001.1182

[36] Yis U, Tufekci UK, Genc S, Carman KB, Bayram E, Topcu Y, et al Expression patterns of micro-RNAs 146a, 181a, and 155 in subacute sclerosing panencephalitis. J Child Neurol. 30 United States: The Author(s) 2014.; 2015 p. 69–74. doi: 10.1177/0883073814531329 24789113 10.1177/0883073814531329

[37] Makeyev EV, Zhang J, Carrasco MA, Maniatis T. The MicroRNA miR-124 promotes neuronal differentiation by triggering brain-specific alternative pre-mRNA splicing. Mol Cell. 2007;27(3):435–48. doi: 10.1016/j.molcel.2007.07.015 . 17679093 10.1016/j.molcel.2007.07.015 PMC3139456

[38] Krichevsky AM, Sonntag KC, Isacson O, Kosik KS. Specific microRNAs modulate embryonic stem cell-derived neurogenesis. Stem Cells. 2006;24(4):857–64. doi: 10.1634/stemcells.2005-0441 . 16357340 10.1634/stemcells.2005-0441 PMC2605651

[39] Silber J, Lim DA, Petritsch C, Persson AI, Maunakea AK, Yu M, et al miR-124 and miR-137 inhibit proliferation of glioblastoma multiforme cells and induce differentiation of brain tumor stem cells. BMC Med. 2008;6:14 doi: 10.1186/1741-7015-6-14 . 18577219 10.1186/1741-7015-6-14 PMC2443372

[40] Li KK, Pang JC, Ching AK, Wong CK, Kong X, Wang Y, et al miR-124 is frequently down-regulated in medulloblastoma and is a negative regulator of SLC16A1. Hum Pathol. 40 United States2009 p. 1234–43. doi: 10.1016/j.humpath.2009.02.003 19427019 10.1016/j.humpath.2009.02.003

[41] Mendrzyk F, Radlwimmer B, Joos S, Kokocinski F, Benner A, Stange DE, et al Genomic and protein expression profiling identifies CDK6 as novel independent prognostic marker in medulloblastoma. J Clin Oncol. 23 United States2005 p. 8853–62. doi: 10.1200/JCO.2005.02.8589 16314645 10.1200/JCO.2005.02.8589

[42] Zheng F, Liao YJ, Cai MY, Liu YH, Liu TH, Chen SP, et al The putative tumour suppressor microRNA-124 modulates hepatocellular carcinoma cell aggressiveness by repressing ROCK2 and EZH2. Gut. 61 England2012 p. 278–89. doi: 10.1136/gut.2011.239145 21672940 10.1136/gut.2011.239145

[43] Furuta M, Kozaki KI, Tanaka S, Arii S, Imoto I, Inazawa J. miR-124 and miR-203 are epigenetically silenced tumor-suppressive microRNAs in hepatocellular carcinoma. Carcinogenesis. 31 England2010 p. 766–76. doi: 10.1093/carcin/bgp250 19843643 10.1093/carcin/bgp250

[44] Yi C, Liu X, Liu Y, Lu S, Qi Y. Hemagglutinin protein of measles virus induces apoptosis of HeLa cells via both extrinsic and intrinsic pathways. Can J Microbiol. 2013;59(12):814–24. Epub 2013/12/10. doi: 10.1139/cjm-2013-0544 . 24313454 10.1139/cjm-2013-0544

[45] Bhaskar A, Bala J, Varshney A, Yadava P. Expression of measles virus nucleoprotein induces apoptosis and modulates diverse functional proteins in cultured mammalian cells. PLoS One. 2011;6(4):e18765 doi: 10.1371/journal.pone.0018765 . 21533140 10.1371/journal.pone.0018765 PMC3077409

[46] Linnstaedt SD, Gottwein E, Skalsky RL, Luftig MA, Cullen BR. Virally induced cellular microRNA miR-155 plays a key role in B-cell immortalization by Epstein-Barr virus. J Virol. 2010;84(22):11670–8. doi: 10.1128/JVI.01248-10 . 20844043 10.1128/JVI.01248-10 PMC2977875

[47] Sun Y, Gui H, Li Q, Luo ZM, Zheng MJ, Duan JL, et al MicroRNA-124 protects neurons against apoptosis in cerebral ischemic stroke. CNS Neurosci Ther. 2013;19(10):813–9. Epub 2013/07/06. doi: 10.1111/cns.12142 . 23826665 10.1111/cns.12142 PMC6493643

[48] Zou D, Chen Y, Han Y, Lv C, Tu G. Overexpression of microRNA-124 promotes the neuronal differentiation of bone marrow-derived mesenchymal stem cells. Neural Regen Res. 2014;9(12):1241–8. doi: 10.4103/1673-5374.135333 . 25206789 10.4103/1673-5374.135333 PMC4146284

[49] Barber GN. Host defense, viruses and apoptosis. Cell Death Differ. 2001;8(2):113–26. Epub 2001/04/21. doi: 10.1038/sj.cdd.4400823 . 11313713 10.1038/sj.cdd.4400823

[50] Roulston A, Marcellus RC, Branton PE. Viruses and apoptosis. Annu Rev Microbiol. 1999;53:577–628. Epub 1999/11/05. doi: 10.1146/annurev.micro.53.1.577 . 10547702 10.1146/annurev.micro.53.1.577

[51] Hay S, Kannourakis G. A time to kill: viral manipulation of the cell death program. J Gen Virol. 2002;83(Pt 7):1547–64. Epub 2002/06/21. doi: 10.1099/0022-1317-83-7-1547 . 12075073 10.1099/0022-1317-83-7-1547

[52] Galluzzi L, Brenner C, Morselli E, Touat Z, Kroemer G. Viral control of mitochondrial apoptosis. PLoS Pathog. 2008;4(5):e1000018 doi: 10.1371/journal.ppat.1000018 . 18516228 10.1371/journal.ppat.1000018 PMC2376094

[53] Nguyen MD, Mushynski WE, Julien JP. Cycling at the interface between neurodevelopment and neurodegeneration. Cell Death Differ. 2002;9(12):1294–306. Epub 2002/12/13. doi: 10.1038/sj.cdd.4401108 . 12478466 10.1038/sj.cdd.4401108

[54] Yoo AS, Staahl BT, Chen L, Crabtree GR. MicroRNA-mediated switching of chromatin-remodelling complexes in neural development. Nature. 2009;460(7255):642–6. doi: 10.1038/nature08139 . 19561591 10.1038/nature08139 PMC2921580

[55] Yeung ML, Bennasser Y, Myers TG, Jiang G, Benkirane M, Jeang KT. Changes in microRNA expression profiles in HIV-1-transfected human cells. Retrovirology. 2005;2:81 doi: 10.1186/1742-4690-2-81 . 16381609 10.1186/1742-4690-2-81 PMC1352379

[56] Triboulet R, Mari B, Lin YL, Chable-Bessia C, Bennasser Y, Lebrigand K, et al Suppression of microRNA-silencing pathway by HIV-1 during virus replication. Science. 315 United States2007 p. 1579–82. doi: 10.1126/science.1136319 17322031 10.1126/science.1136319

[57] Chiang K, Liu H, Rice AP. miR-132 enhances HIV-1 replication. Virology. 2013;438(1):1–4. doi: 10.1016/j.virol.2012.12.016 . 23357732 10.1016/j.virol.2012.12.016 PMC3594373

[58] Bhanja Chowdhury J, Shrivastava S, Steele R, Di Bisceglie AM, Ray R, Ray RB. Hepatitis C virus infection modulates expression of interferon stimulatory gene IFITM1 by upregulating miR-130A. J Virol. 2012;86(18):10221–5. doi: 10.1128/JVI.00882-12 . 22787204 10.1128/JVI.00882-12 PMC3446586

[59] Avila-Bonilla RG, Yocupicio-Monroy M, Marchat LA, De Nova-Ocampo MA, Del Angel RM, Salas-Benito JS. Analysis of the miRNA profile in C6/36 cells persistently infected with dengue virus type 2. Virus Res. 232 Netherlands: 2017 Elsevier B.V; 2017 p. 139–51. doi: 10.1016/j.virusres.2017.03.005 28267608 10.1016/j.virusres.2017.03.005

[60] Jopling C. Liver-specific microRNA-122: Biogenesis and function. RNA Biol. 2012;9(2):137–42. doi: 10.4161/rna.18827 . 22258222 10.4161/rna.18827 PMC3346312

[61] Jopling CL, Yi M, Lancaster AM, Lemon SM, Sarnow P. Modulation of hepatitis C virus RNA abundance by a liver-specific MicroRNA. Science. 309 United States2005 p. 1577–81. doi: 10.1126/science.1113329 16141076 10.1126/science.1113329

[62] Villanueva RA, Jangra RK, Yi M, Pyles R, Bourne N, Lemon SM. miR-122 does not modulate the elongation phase of hepatitis C virus RNA synthesis in isolated replicase complexes. Antiviral Res. 2010;88(1):119–23. doi: 10.1016/j.antiviral.2010.07.004 . 20637242 10.1016/j.antiviral.2010.07.004 PMC4422393

[63] Roberts AP, Lewis AP, Jopling CL. miR-122 activates hepatitis C virus translation by a specialized mechanism requiring particular RNA components. Nucleic Acids Res. 2011;39(17):7716–29. doi: 10.1093/nar/gkr426 . 21653556 10.1093/nar/gkr426 PMC3177192

[64] Israelow B, Mullokandov G, Agudo J, Sourisseau M, Bashir A, Maldonado AY, et al Hepatitis C virus genetics affects miR-122 requirements and response to miR-122 inhibitors. Nat Commun. 2014;5:5408 doi: 10.1038/ncomms6408 . 25403145 10.1038/ncomms6408 PMC4236719

[65] McQuaid S, Campbell S, Wallace IJ, Kirk J, Cosby SL. Measles virus infection and replication in undifferentiated and differentiated human neuronal cells in culture. J Virol. 1998;72(6):5245–50. . 9573298 10.1128/jvi.72.6.5245-5250.1998 PMC110109

